# Implementation science in resource-poor countries and communities

**DOI:** 10.1186/s13012-018-0847-1

**Published:** 2018-12-27

**Authors:** H. Manisha Yapa, Till Bärnighausen

**Affiliations:** 1 The Kirby Institute, University of New South Wales, Sydney, Australia; 2 Africa Health Research Institute (AHRI), KwaZulu-Natal, South Africa; 3 Department of Global Health and Population, Harvard T.H. Chan School of Public Health, Boston, USA; 4 Heidelberg Institute of Global Health, Medical Faculty and University Hospital, University of Heidelberg, INF 130.3, 69120 Heidelberg, Germany

**Keywords:** Implementation, Resource-poor settings, Resources, Capacity, Reverse innovation, Research methods, Capacity building

## Abstract

**Background:**

Implementation science in resource-poor countries and communities is arguably more important than implementation science in resource-rich settings, because resource poverty requires novel solutions to ensure that research results are translated into routine practice and benefit the largest possible number of people.

**Methods:**

We reviewed the role of resources in the extant implementation science frameworks and literature. We analyzed opportunities for implementation science in resource-poor countries and communities, as well as threats to the realization of these opportunities.

**Results:**

Many of the frameworks that provide theoretical guidance for implementation science view resources as contextual factors that are important to (i) predict the feasibility of implementation of research results in routine practice, (ii) explain implementation success and failure, (iii) adapt novel evidence-based practices to local constraints, and (iv) design the implementation process to account for local constraints. Implementation science for resource-poor settings shifts this view from “resources as context” to “resources as primary research object.” We find a growing body of implementation research aiming to discover and test novel approaches to generate resources for the delivery of evidence-based practice in routine care, including approaches to create higher-skilled health workers—through tele-education and telemedicine, freeing up higher-skilled health workers—through task-shifting and new technologies and models of care, and increasing laboratory capacity through new technologies and the availability of medicines through supply chain innovations. In contrast, only few studies have investigated approaches to change the behavior and utilization of healthcare resources in resource-poor settings. We identify three specific opportunities for implementation science in resource-poor settings. First, intervention and methods innovations thrive under constraints. Second, reverse innovation transferring novel approaches from resource-poor to research-rich settings will gain in importance. Third, policy makers in resource-poor countries tend to be open for close collaboration with scientists in implementation research projects aimed at informing national and local policy.

**Conclusions:**

Implementation science in resource-poor countries and communities offers important opportunities for future discoveries and reverse innovation. To harness this potential, funders need to strongly support research projects in resource-poor settings, as well as the training of the next generation of implementation scientists working on new ways to create healthcare resources where they lack most and to ensure that those resources are utilized to deliver care that is based on the latest research results.

Many of the physical constraints that impede the routine delivery of effective health interventions to those who can benefit are (by definition) far more severe in resource-poor than in resource-rich countries. For instance, for each citizen, the resource-poor countries of sub-Saharan Africa spend only a fraction of the amount on health that the resource-rich countries of Western Europe spend, and the numbers of doctors and nurses per population are orders of magnitudes lower in Africa than in Europe (Fig. 1). At the same time, amenable mortality—i.e., the mortality that existing effective healthcare technologies could eliminate if they were delivered successfully to all those who can benefit—is far higher in resource-poor countries than in resource-rich ones (Fig. 1) [1, 2]. This “inverse care law” in cross-country comparison—the “availability of good medical care tends to vary inversely with the need for it in the population served” [3]—is of course merely a global version of the classic inverse care law, which operates across communities within both resource-rich and resource-poor countries. In this editorial, we are addressing specific features of implementation science for both resource-poor countries and resource-poor communities, recognizing that scarcity and deprivation affecting the delivery of evidence-based healthcare exist worldwide and across all geographic areas and that there is a continuum from resource poverty to resource wealth in all countries.

**Fig. 1:**
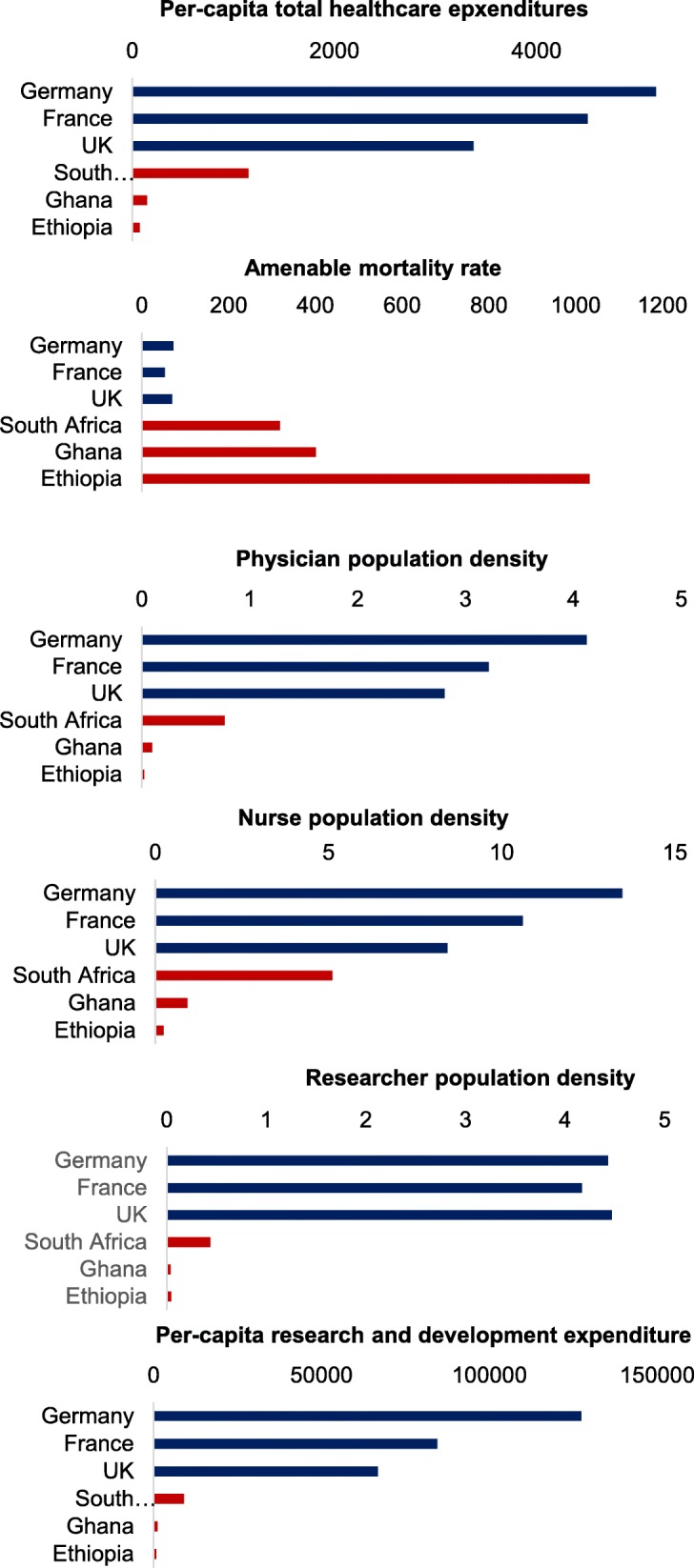
Comparing resource-rich and resource-poor countries. Per-capita total healthcare expenditures and per-capita research and development expenditures are in 2011 international $. Physician, nurse, and researcher population densities are shown per 1000 population

An obvious approach to reduce the high levels of amenable mortality in resource-poor countries and communities is to increase the financial resources available for healthcare. This approach, however, requires either substantial economic growth—which may fail to emerge in both resource-poor countries [4] and communities [5]—a redistribution of existing resources across sectors—which is difficult to achieve for obvious political reasons [6]—or external assistance—which cannot be relied on over the long term as donor priorities shift frequently [7, 8]. Another approach is to create new resources to deliver effective health interventions given the existing financial constraints. Implementation science can contribute to this approach as the science of the discovery, design, and evaluation of novel approaches to deliver evidence-based healthcare practice.

## Creating resources

The goal of implementation science is to discover and test approaches “to promote the systematic uptake of research findings and other evidence-based practices into routine practice, and, hence, to improve the quality and effectiveness of health services” [9]. Many of the frameworks that provide theoretical guidance for implementation science feature resources and physical capacity to deliver evidence-based practice—such as health workers, drugs, supply chains, and healthcare facilities—as part of the context of implementation [10–27]. In these frameworks, assessments of the resources context are used to guide analysis or action, e.g., to (i) predict the feasibility of implementation of a novel evidence-based practice [16, 25, 28], (ii) explain implementation success and failure [11–13, 24, 26, 29], (iii) adapt a novel evidence-based practice to local constraints [15, 19, 20, 23, 30], and (iv) design the implementation process to account for local constraints [17, 22, 30]. As such, in these theoretical frameworks—and in the implementation science for resource-rich settings they have been derived from and guide—resources are viewed as important contextual factors. Implementation science for resource-poor settings shifts this view from “resources as context” to “resources as primary research object” [31]. Table 1 shows examples of implementation science in resource-poor countries and communities testing approaches to expand human resources for health—through tele-education, telemedicine, task-shifting to lower-skilled health workers, task-shifting to clients, new models of care, and technological innovation—and to increase laboratory capacity and supplies. A large body of implementation science in resource-poor countries and communities has focused on creating resources for evidence-based healthcare. This research is likely to continue with vigor because “there need to be minimal human resources, financing, drugs, and supply systems before effective interventions can be delivered” [31]. In particular, research developing and testing community health worker programs [32]—which are widely viewed as one of the few viable solutions to the persistent health worker shortages in many resource-poor countries and communities [33–35]—and information and communication technologies—which can provide affordable training and decision support for health workers anywhere—will continue to attract increasing implementation research funding [36–38].

**Table 1:** Implementation research to increase resources

Health systems function	Delivery innovation	Delivery control	Outcomes	Study design	Population	Country	Reference
Creating higher-skilled human resources for health through tele-education							
Training on neonatal resuscitation	Tele-education	Conventional classroom teaching	• Knowledge scores • Skills scores	RCT	Staff nurses	India	Jain et al. *Journal of Perinatology* 2010 [112]
Training on retinopathy of prematurity diagnosis	Tele-education	Standard onsite training	• Sensitivity of retinopathy of prematurity diagnosis • Specificity of retinopathy of prematurity diagnosis	RCT	Ophthalmology residents	Mexico	Patel et al. *Ophthalmology* 2017 [113]
Education on nursing, public health, child and adolescent health, mental health	Tele-education	No control	• Tele-education participation • User satisfaction	Process evaluation	Primary care staff	Brazil	Joshi et al. *Journal of Telemedicine and Telecare* 2011 [114]
Creating higher-skilled human resources for health through telemedicine							
Endocrine surgery	Telemedicine (tele-education and surgical treatment planning, teleconsultation, telepathology, teleradiology, and telesurgical conferences)	Standard of care at the time of the study	• Endocrine surgery rate	UBA	General surgeons	India	Pradeep et al. *World Journal of Surgery* 2007 [115]
Dermatological diagnosis	Internet-based teledermatology system	Face-to-face examination	• Agreement between the two diagnostic approaches	Validation study	Junior doctors	Brazil	Chao et al. *Telemedicine Journal and Ehealth* 2003 [116]
Intensive care	Tele-intensive care unit	Standard of care at the time of the study	• Number of ICU patients per month	UBA	Nurses	Syria	Moughrabieh et al. *Annals of the American Thoracic Society* 2016 [117]
Freeing up human resources through task-shifting to lower-skilled health workers							
HIV treatment initiation and management	Nurses	Standard of care at the time of the study (doctors)	• Mortality • Viral suppression	RCT	Adult HIV patients in primary care	South Africa	Fairall et al. *Lancet* 2012 [118]
Depression and anxiety screening, diagnosis and treatment	Lay village health workers together with primary care doctors, supported by an electronic decision support system	Standard of care at the time of the study (trained mental health professionals)	• Coverage with mental health treatment • Depression score • Anxiety score	UBA	Members of rural scheduled tribe communities	India	Maulik et al. *Journal of Global Health* 2017 [119]
Hypertension treatment	Community health nurses delivering the WHO Package of Essential NCD Interventions (WHO PEN)	Standard of care at the time of the study	• Blood pressure	RCT	Patients visiting community health centers	Ghana	Ogedegbe et al. *Implementation Science* 2014 [120]
HIV and HIV risk screening and linkage to care for children	Community health workers	Standard of care at the time of the study	• Identification of HIV-infected and HIV-exposed children • Linkage to care	UBA	Children born to mothers living with HIV	Malawi	Ahmed et al. *Journal of the International AIDS Society* 2015 [121]
HIV treatment	Lay health workers	Standard of care at the time of the study (doctors and nurses)	• Viral suppression	RCT	Adult HIV patients in primary care	Tanzania	Geldsetzer et al. *BMC Health Services Research* 2017 [122]
Antenatal and postnatal counseling	Lay nurse aides using job aids	Professional nurses using job aids	• Coverage with correct antenatal and postnatal messages • Pregnant women with correct antenatal knowledge	NRC	Women in antenatal care	Benin	Jennings et al. *Implementation Science* 2011 [123]
HIV treatment initiation	Community health workers providing home-based HIV treatment initiation	Standard of care at time of the study (only facility-based initiation of HIV treatment)	• HIV treatment initiation	RCT	General population	Malawi	Macpherson et al. *JAMA* 2014 [124]
Freeing up human resources through task-shifting to clients							
HIV testing	HIV self-testing	Standard of care at the time of the study (facility HIV testing)	• HIV testing rates	RCT	Female sex workers	Uganda, Zambia	Ortblad et al. *PLOS Medicine* 2017 [125], Chanda et al. *PLOS Medicine* 2017 [126]
HIV testing	Unsupervised HIV self-testing	Provider-supervised HIV self-testing	• Sensitivity	RCT	Fisherfolk	Uganda	Asiimwe et al. *AIDS & Behavior* 2014 [127]
Cervical cancer screening	Vaginal self-collection of specimens	Cervical specimens collection by clinician	• Sensitivity • Specificity	Validation study	Adult women	India, Nicaragua, Uganda	Jeronimo et al. *International Journal of Gynecological Cancer* 2014 [128]
Freeing up human resources through new models of care							
HIV treatment	Community-based adherence clubs	Standard of care at the time of the study	• Loss to follow-up • Viral suppression	NRC	Adult HIV patients in primary care	South Africa	Grimsrud et al. *JAIDS* 2016 [62]
Buruli ulcer detection and treatment	Buruli ulcus community of practice composed of hospital staff, former patients, CHWs, and traditional healers	Standard of care at the time of the study	• Buruli ulcus detection rate • Buruli ulcus treatment adherence	UBA	General population	Cameroon	Awah et al. *PLOS Neglected Tropical Diseases* 2018 [129]
Family healthcare services	Community-based family health program	Standard of care at the time of the study	• Mortality rates • Causes of death • Adult employment • School enrollment	UBA	Children (aged 10–17) and adults	Brazil	Rocha et al. *Health Economics* 2010 [130]
Freeing up human resources through technological innovations							
Encouragement to remain in postpartum care	Text messages	Standard of care at the time of the study	• Maternal postpartum visit attendance • Early infant HIV testing	RCT	Pregnant women enrolled in public sector PMTCT program	Kenya	Odeny et al. *AIDS* 2014 [131]
Hypertension treatment	Automated self-management calls plus home blood pressure monitoring	Standard of care at the time of the study	• Systolic blood pressure	RCT	Adult patients with hypertension in primary care	Honduras and Mexico	Piette et al. *Telemedicine Journal and Ehealth* 2012 [132]
Encouragement to adhere to hypertension treatment	Text messages	Standard of care at the time of the study	• Systolic blood pressure	RCT	Adult patients with hypertension in primary care	South Africa	Bobrow et al. *Circulation* 2016 [133]
Encouragement to adhere to HIV treatment	Text messages	Standard of care at the time of the study	• Adherence • Viral suppression	RCT	Adult patients with hypertension in primary care	Kenya	Lester et al. *Lancet* 2010 [134]
Neurocognitive impairment screening	NeuroScreen mobile app administered by a lay health worker	Neuropsychological test battery administered by research psychometrist	• Sensitivity • Specificity	Validation study	Adult HIV patients in primary care	South Africa	Robbins et al. *Journal of Medical Internet Research Mhealth Uhealth* 2018 [77]
Increasing laboratory capacity through technological innovations							
Viral load monitoring	Point-of-care viral load test using capillary blood	Laboratory viral load test using venous blood	• Sensitivity • Specificity	Validation study	Adult HIV patients in primary care	Mozambique	Jani et al. *Journal of Clinical Microbiology* 2016 [135]
CD4 testing	Point-of-care CD4 test using capillary blood	Laboratory CD4 test using venous blood	• Sensitivity • Specificity	Validation study	Adult HIV patients in primary care	Zimbabwe	Mtapuri-Zinyowera et al. *Journal of Acquired Immune Deficiency Syndromes* 2010 [136]
CD4 testing	Point-of-care CD4 test using capillary blood	Laboratory CD4 test using venous blood	• Loss to follow-up	UBA	Adult HIV patients in primary care	Mozambique	Jani et al. *Lancet* 2011 [137]
Tuberculosis diagnosis	Point-of-care TB test performed by nurses in primary care clinics	Laboratory TB test	• Sensitivity • Specificity • Same-day diagnosis • Same-day treatment initiation • Loss to follow-up	cRCT	Adult primary care patients	South Africa, Tanzania, Zambia, Zimbabwe	Theron et al. *Lancet* 2014 [138]
Breast cancer screening	Point-of-care breast imaging device	Standard of care (clinical breast examination)	• Sensitivity • Specificity • Positive predictive value • Negative predictive value	Validation study	Healthy women visiting a hospital	India	Somashekar et al. *Indian Journal of Gynecologic Oncology* 2016 [139]
Increasing the availability of medicines through supply chain innovations							
Nevirapine (NVP) prophylaxis for HIV-exposed infants	Pratt Pouch delivery system	No control	• Administration of NVP to infants • Infant dried blood spot NVP concentration	Process evaluation	HIV-exposed infants and their mothers	Tanzania	Dahinten et al. *Pediatric Infectious Diseases* 2016 [140]
Access to artemisinin-based combination therapy (ACT) antimalarials	Private-sector Accredited Drug Dispensing Outlet (ADDO)	Public sector distribution	• Uptake of ACT • Availability of ACT	UBA	Adults and children	Tanzania	Rutta et al. *Health Research Policy and Systems* 2011 [141]
Access to oral rehydration salts (ORS) and zinc for children	Private-sector distribution channels (Coca Cola)	Public sector distribution	• Availability of ORS and zinc at rural retail outlets • Distance traveled by caregivers to obtain ORS and zinc • Use of ORS and zinc in infants	CBA	Community retailers, children and their caregivers	Zambia	Berry et al. *Endline report: Colalife Operational Trial Zambia* 2014 [142]
Vaccine supply chain	Public-private partnership for vaccine supply	Government-managed supply	• Vaccine stock • Immunization coverage	UBA	Regional zone stores, primary healthcare facilities	Nigeria	Molemodile et al. *Global Public Health* 2017 [143]
Supply of health workers, essential medicines and equipment to remote villages	Systematic motorcycle fleet management for health care supplies (supply of high-quality motorcycles, driver training, preventive maintenance, fuel, on demand repair)	Standard of care motorcycle fleet management for health care supplies	• Trips to rural villages per health worker per week • Patient visits per health worker per week • Measles immunization per health worker per week • Child growth assessment per health worker per week	CBA	Village health workers	Zambia	Mehta et al. *American Journal of Public Health* 2015 [144]

*RCT* randomized controlled trial, *UBA* uncontrolled before-after study, *CBA* controlled before-after study, *NRC* non-randomized controlled study, *WSuV* within-subject validation study, *PMTCT* prevention of mother-to-child transmission of HIV program

## Changing behavior

In contrast to research aimed at increasing resources, to date, comparatively few studies in resource-poor settings have investigated approaches to change the behavior and utilization of those resources to ensure that research findings are translated into routine practice. A 2017 “overview of systematic reviews” on “implementation strategies for health systems in low-income countries” published in the *Cochrane Database of Systematic Reviews* is a case in point [39]. The 18 systematic reviews on different strategies to change health worker behavior in this overview article—education materials [40], internet-based learning [41], educational meetings and workshops [42–45], educational outreach [46–48], local opinion leaders [49], audit and feedback [50], reminders [51], tailored interventions [52], and multi-faceted interventions [42, 47, 50, 53]—synthesized 820 primary studies. Among these primary studies, which can be viewed as the global knowledge base on strategies to change health worker behavior, only 13 (or 1.6%) took place in a low-income country and only 82 (10.0%) took place in a middle-income country. There is thus strong potential for resource-poor countries to learn from the experiences in resource-rich countries. Clearly, some evidence generated in resource-rich settings is highly relevant for resource-poor settings—if “the implementation strategies considered … address a problem that is important in low-income countries, would be feasible, and would be of interest to decision-makers in low-income countries” [39]. Equally clearly, however, studies systematically investigating the transferability of the large body of evidence on strategies to change health worker behavior generated in resource-rich countries are urgently needed. In addition to the obvious resources gradient, reasons why evidence on effective practice cannot be transferred from resource-rich to resource-poor settings may include important differences in political and institutional factors [54–56]. While transfer of evidence from any one to any other context will always need to take account of these factors, there will often be particularly large differences in the answers to questions such as those posed by the “Tailored Implementation for Chronic Diseases Checklist” (TICD Checklist) when considering evidence transfer from resource-rich to resource-poor settings: Do “influential people”, “political stability”, and “corruption” “facilitate or hinder implementation of necessary changes?” [30]. In many cases, successful implementation of evidence-based practice in resource-poor settings will thus require research to learn how to best adopt strategies that have proven effective in resource-rich settings, as well as the discovery and evaluation of wholly new approaches.

## Creativity and reverse innovation

Resource constraints, however, are not only an important object of implementation research in resource-poor countries and communities, but they are also a powerful stimulus for creativity [57]. The psychological and marketing literature shows that creativity thrives when choices are restricted [58–60]. It is likely that the severe human and physical resources constraints in the health systems of resource-poor countries and communities have boosted discovery in implementation science for health. Routine healthcare in resource-poor countries and communities is often provided by nurses and community health workers, without access to basic medical equipment, in primary care clinics or in homes without reliable referral chains to higher-level care. As a result of these constraints and the large differences between “ideal” and “real-world” delivery in resource-poor countries and communities, innovation is likely to thrive, because greater creativity is required to ensure that scientific innovations can be delivered in routine healthcare practice.

The implementation research leading to novel approaches to deliver HIV care in resource-poor countries and communities illustrates this creativity. Implementation researchers have worked with implementers to discover, design, and test such highly innovative approaches as social clubs [61–66], street dispensing machines [67, 68], and drones [69, 70] to deliver HIV antiretroviral drugs, as well as mobile phone technology to provide HIV prevention education [71–73]. In many other areas, major and minor innovations are continuously increasing capacity and quality of care in resource-poor countries and communities, such as the multitude of novel eHealth [74, 75], mHealth [76–79], and telemedicine [80] applications. This creativity under constraints leads to potential for “reverse innovation” [81, 82], i.e., innovation arising first in resource-poor settings and only later spreading to resource-rich settings. According to a recent review, important areas for future “reverse innovation” in healthcare include “rural health service delivery; skills substitution; decentralisation of management; creative problem-solving; education in communicable disease control; innovation in mobile phone use; low technology simulation training; local product manufacture; health financing; and social entrepreneurship” [83]. In several research areas—e.g., skills substitution and innovation in mobile phone use (Table 1)—evidence is likely to continue to increase substantially in resource-poor—but not in resource-rich—settings, opening up opportunities for “reverse” flows of innovation and experience.

## Methods innovations

The definitional characteristic of resource-poor settings, resource poverty, also has implications for the methods of implementation science, stimulating the development of new approaches. For instance, the stepped-wedge cluster randomized controlled trial—in which clusters, such as communities or clinics, are randomized to an exposure sequence over time rather than to one time-invariant exposure as in the traditional parallel-arm trial—was first envisioned, developed, and used for a study in The Gambia in 1987 [84]. The stepped-wedge trial remains a methods mainstay of implementation science in resource-poor countries today [85–89]. One of the motivations for choosing a stepped-wedge over a parallel-arm design is that in the latter all communities “within the study eventually receive the intervention, thereby improving equity and acceptability” [90]. In contrast, traditional parallel-arm cluster randomized trials withhold the intervention that is tested from the communities in the control arm over the entire course of the study. This assignment can lead to political opposition to a study, because community members perceive value in the intervention to be tested. Such political opposition, in turn, is typically stronger in resource-poor than in resource-rich communities, because the former often lack many of the basic amenities and services that the latter have good access to.

Other methods innovations in implementation science in resource-poor countries have been driven by a lack of resources for science. On average, low-income countries spend far less money on science and have far fewer scientists per population than high-income countries [91] (Fig. 1). To overcome resource constraints in research, implementation scientists have developed novel approaches to collect and analyze data using information and communication technologies. These innovations include field workers and community health workers using mobile phones to collect survey data [92], screen for diseases [93], and record healthcare utilization events [94].

Resource poverty can also cause or exacerbate variation in the scale-up of novel interventions across communities and—because of rationing—across individuals [95]. Such exposure variations, in turn, offer opportunities for innovative quasi-experiments to evaluate implementations of health interventions. Examples of such quasi-experimental designs include regression discontinuity—which can be used when threshold rules are used to determine eligibility for an intervention [96, 97]—and difference-in-differences—which exploits changes in intervention exposure in one set of communities while the exposure in another set remains unchanged [98, 99]. Quasi-experiments have the added advantage that they are typically far cheaper to carry out than experiments which require a prospective research infrastructure and substantial investment in trial processes. Finally, quasi-experiments take place in “real-life” without the distorting influences of experimental intervention which can introduce artificiality into the implementation context [100]. As such, quasi-experiments have been popular to establish causal impacts of interventions in resource-poor countries and communities [101], but they are of course equally valuable in resource-rich settings [102].

## Creating research capacity

Implementation science is unlikely to be an exception to the general rule that resource-poor countries have far fewer researchers per population than resource-rich countries (Fig. 1). It may be possible to overcome the resulting “inverse care law” of implementation science—capacity is lowest where need is highest—with innovative solutions for training the next generation of implementation researchers in resource-poor countries. Major international funders, such as the Fogarty International Center of the US National Institutes of Health, are currently making large investments in South-South and South-North partnerships for implementation science training [103]. Several universities in the Global South have recently started to offer master and doctoral degrees in implementation science, such as the University of Nairobi (Kenya), University of Ghana, University of Zambia, University of the Witwatersrand (South Africa), BRAC University (Bangladesh), Universidad de Antioquia (Colombia), Universitas Gajdah Mada (Indonesia), and the University of Beirut (Lebanon) [104]. Another important opportunity to increase capacity for implementation science are massive open online courses (MOOCs), which provide (free or inexpensive) training in implementation science through online learning platforms (see Table 2 for two examples). Reflecting the reality of implementation science projects in resource-poor countries, these research programs include training in theory and formative research for intervention design; process, impact, and economic evaluation methods; and approaches for knowledge dissemination and policy translation. Despite these promising initiatives, the availability of researchers in resource-poor countries who have been rigorously trained in quantitative, qualitative, and mixed methods for implementation research remains low [105].

**Table 2:** Massive open online courses in implementation science

Course	Organization	Duration	Content
Fundamentals of Implementation Science	University of Washington, USA	11 weeks	• Relevance of implementation science to global health • Impact evaluation methods • Economic analysis methods • Stakeholder and policy analysis • Qualitative health systems research • Quality improvement as a management tool • Disseminating research findings
Specialist Certificate in Implementation Science	University of Melbourne, Australia	6 months	• Conceptual models and frameworks • Role of data in driving implementation success • Different approaches to implementation • Process evaluation • Formative research • Outputs and outcomes • Impact evaluation

## Science for policy

An important counterpoint to the triad of high need, high potential, and low capacity for implementation science in resource-poor countries and communities is the powerful opportunities for policy impact that engagement with policy makers offer. In many resource-poor countries, policy makers and stakeholders are closely involved in implementation research, ranging from the conception of research ideas to the interpretation of findings and from leading research agenda setting exercises with scientists [106, 107] to principal investigator roles in scientific studies [87]. Close collaboration between implementation scientists and policy makers is not constrained to resource-poor settings [108], but it is likely particularly strong in those settings because of the higher need for implementation evidence when the capacity to deliver interventions is extremely scarce as well as a culture of testing the delivery of scientific innovations in “demonstration projects” to guide policy decisions and the design for long-term routine practice. For instance, many African countries are currently considering adopting HIV pre-exposure prophylaxis (PrEP) as routine health policy but are unsure which delivery models work best in their specific contexts. To fill this knowledge gap, more than 50 PrEP demonstration projects in Africa are currently experimenting with alternative delivery models [109, 110].

## Conclusion

In any setting, the results of implementation science can lead to improved routine healthcare practice. In resource-poor countries and communities, however, the need for such results is arguably higher than in resource-rich countries, while the capacity to carry out implementation research is lower. Despite this “inverse care law of implementation science,” several specific opportunities for implementation science in resource-poor settings exist. First, intervention and methods innovations thrive under constraints. Second, reverse innovation transferring novel approaches from resource-poor to research-rich settings will gain in importance. Third, policy makers in resource-poor countries tend to be interested in collaborating closely with scientists on implementation research projects aimed at informing national and local policy. To realize these opportunities, several actions are needed. Funders need to increase their commitments to implementation science in resource-poor settings [111]. Funders and universities need to increase their investment in training the next-generation of implementation scientists who devote their careers to discovering and testing novel approaches to create and influence healthcare resources where they lack most. Finally, journal editors need to signal strongly that they are interested in featuring results from rigorous implementation science originating in resource-poor settings, to ensure that some of the brightest graduate students can be recruited into this field. The results of such actions will likely lead to a double benefit—generating major scientific advances and contributing to improved health among the world’s poor.
